# Prevalence of vitiligo among patients attending the skin clinic at Mbarara Regional Referral Hospital, southwestern Uganda: A retrospective review

**DOI:** 10.1016/j.jdin.2025.01.010

**Published:** 2025-02-20

**Authors:** Simon Peter Mundeli, Aloyo Gladys Onguti, Mirembe Stephen Kizito, Mulyowa Grace Kitunzi, Timothy Obrien

**Affiliations:** a Department of Dermatology, Mbarara University of Science and Technology (MUST), Mbarara city, Uganda; b Department of Dermatology, St. Vincent’s Hospital and Peter Macallum Cancer Center, Melbourne, Australia

**Keywords:** Africa, epidemiology, incidence, Mbarara Regional Referral Hospital, Mbarara University of Science and Technology, prevalence, Uganda, vitiligo

*To the Editor:* Vitiligo is a chronic skin disorder characterized by the loss of melanocytes, resulting in depigmented macules and patches. It affects 0.1% to 2% of the global population^1^ and occurs equally in both genders, often presenting in early life. Despite its global prevalence, data on vitiligo in Uganda are limited, posing challenges for effective health care planning and management. This gap is further exacerbated by a critical shortage of specialist dermatologists in Uganda, with only 12 serving a population of over 45 million.

A cross-sectional hospital-based study that retrospectively reviewed medical records of patients attending the skin clinic from January 1st to December 31st, 2023, was conducted. The Mbarara Regional Referral Hospital (MRRH) skin clinic was selected for its extensive catchment area, serving over 4 million people in southwestern Uganda.

The inclusion criteria were adequately completed and detailed patient records from the year 2023, while the exclusion criteria included incomplete or duplicate records and records from years other than 2023.

Data were collected from the Health Management Information System registers under a consent waiver, and the collection tool captured the number of vitiligo cases, total clinic visits during the year, and their demographic details.

The data were verified for completeness and analyzed using Stata version 17.0. Prevalence was calculated as the proportion of vitiligo cases among all clinic attendees during the study period.

A waiver of consent was obtained to access patient records while maintaining confidentiality and privacy through data deidentification.

Of the 3444 new patients at MRRH’s skin clinic in 2023, 61 were diagnosed with vitiligo, resulting in a prevalence of 1.77%. The median age was 24 years, with a slight female predominance (54%) as detailed in Table I.

**Table I:** Descriptive characteristics of patients with vitiligo and classification of the various forms of vitiligo diagnosed

Variable	Total (*N* = 61)
Age	24 (13-36)
Sex	
F	33 (54%)
M	28 (46%)
Vitiligo classification	
Generalized vitiligo	
Vitiligo vulgaris	43 (70%)
Vitiligo universalis	2 (3%)
Localized vitiligo	
Focal vitiligo	
Genital vitiligo	4 (7%)
Other	6 (10%)
Segmental vitiligo	3 (5%)
Variants	
Trichrome vitiligo	1 (2%)
Vitiligo stability	
Unstable vitiligo	2 (3%)

Population-based studies in the United States and China reported vitiligo prevalence of 0.76%^2^ and 0.093%,^3^ respectively. These prevalence values are generally lower than in our hospital-based study, which reported a slightly higher vitiligo prevalence of 1.77%, comparable to the 1.2% found in a hospital-based study in Senegal.^4^ Females were slightly more affected than males, with a ratio of 1.2:1, similar to findings from the Regional Dermatology Training Centre in Moshi, Tanzania, where women accounted for 63.9% of cases.^5^ The similarities in vitiligo gender prevalence between southwestern Uganda and Moshi, Tanzania, may be influenced by genetic factors.

In our study, 70% of patients had vitiligo vulgaris. Among those with localized vitiligo, 7% were children with the focal genital type as detailed in Fig 1. Only 1 patient was documented with the trichrome variant of vitiligo, indicating challenges with vitiligo variant documentation.

**Fig 1:**
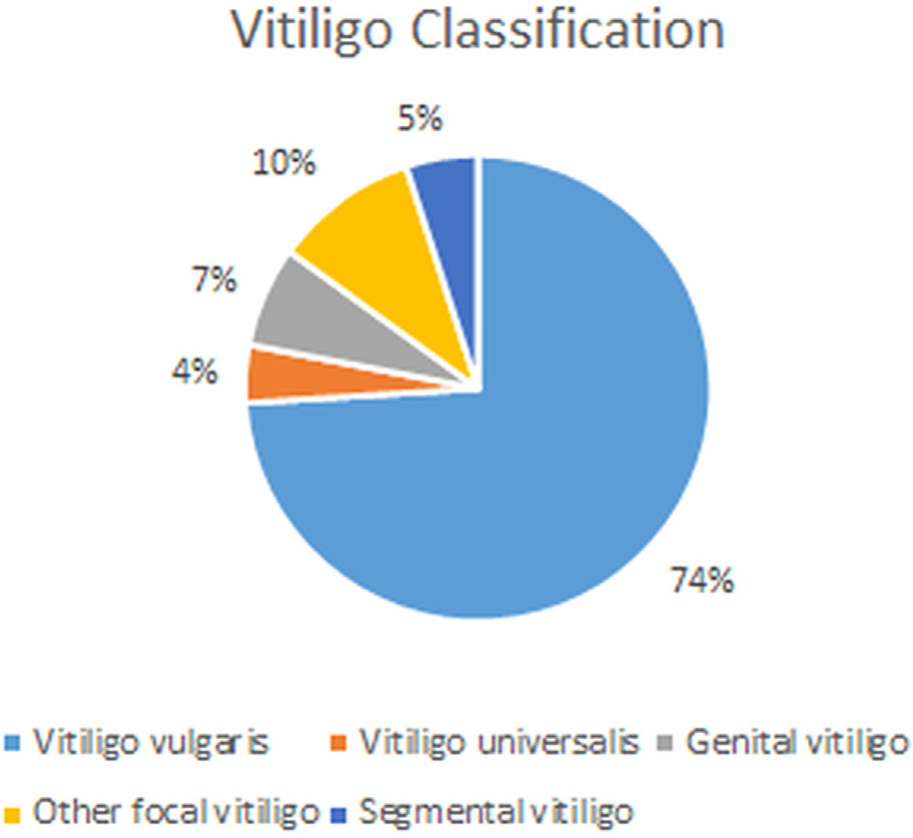
Vitiligo classification showing the percentages of the generalized and localized types of vitiligo obtained from the medical records.

This study found a vitiligo prevalence of 1.77% among patients at MRRH's skin clinic. The need for enhanced clinical documentation of vitiligo variants, including details on vitiligo stability, was highlighted to improve patient management and resource allocation in dermatological care in Uganda.

Limitations of this study include incomplete or missing data, selection bias, single-center design, and potential misdiagnosis.

## Conflicts of interest

None disclosed.
